# Lipoma Causing Death1Read before the meeting of the Wisconsin Society of Veterinary Graduates, Madison, February 5, 1897.

**Published:** 1897-05

**Authors:** J. F. Roub

**Affiliations:** Monroe, Wis.


### REPORTS OF CASES.

### LIPOMA CAUSING DEATH.[1]

By J. F. Roub, D.V.S.,

MONROE, WIS.

The patient was a dark-brown gelding, sixteen hands high, and nineteen years of age, weighing 1200 pounds, and used for a carriage-horse; never known to refuse his food and never had a sick day previous to the time I was called to see him. Upon examination I found the pulse almost imperceptible; temperature 103° F.; perspiration bedewed the cutaneous surface; visible mucous membranes somewhat injected; peristalsis normal; and the feces were normal in appearance. The horse was suffering excruciating pain.

[1] Read before the meeting of the Wisconsin Society of Veterinary Graduates, Madison, February 5, 1897.

The pain was continuous and presented the characteristic symptoms of enteritis, and I informed the owner that his horse would probably die. It being only a short distance from my hospital, I had the animal taken there. This was at 8 A.M. I began treatment by giving opiates and anodynes in large doses, but all to no avail. The horse continued to roll and thresh about until he was nearly exhausted. At 1.30 P.M. I gave morphine sulph., gr. v., hypodermatically. At this time I informed the owner that I was mistaken in my diagnosis, as it would be impossible for the horse to have enteritis and peristalsis continued for five hours and a half. I then made a rectal examination, but discovered nothing that would lead to a true diagnosis. Being at a loss as to the cause, I waited for further developments. At 2 P.M. the horse grew quieter and seemed almost free from pain. At 3 P.M. he rolled a few times, and then lay in a recumbent position, partly comatose, for about four hours. At 8 P.M. pulse imperceptible; a cold sweat bedewed the body; extremities cold; haggard expression of the countenance; tremor of pectoral muscles; staggering gait; temperature 98° F.; intestines paralyzed. At this point I was sure I had a case of enteritis, but did not inform the owner. There was not much change in the symptoms until 8 A.M., when the patient grew much worse and died at 9 A.M.

The animal was taken out two and one-half miles, and the autopsy revealed a lipomatous tumor resembling a large apple, measuring four and one-half inches in diameter; the pedicle was three-eighths of an inch in diameter, being attached to the posterior aorta three inches posterior to the lesser mesenteric artery, causing pressure on the aorta sufficient to diminsh the calibre of the vessel three-fourths. This caused infiltration, and there was a black, gelatinous mass, varying from one-half to one inch in thickness and covering a surface of twelve to fifteen inches, also extending down the lesser mesentery about eight inches. The intestinal tract and all the other internal organs were in a healthy condition.

